# Comprehensive Multiple Molecular Profile of Epithelial Mesenchymal Transition in Intrahepatic Cholangiocarcinoma Patients

**DOI:** 10.1371/journal.pone.0096860

**Published:** 2014-05-09

**Authors:** Xiao-Yong Huang, Chi Zhang, Jia-Bin Cai, Guo-Ming Shi, Ai-Wu Ke, Zhao-Ru Dong, Peng-Fei Zhang, Jia Fan, Bao-Gang Peng, Jian Zhou

**Affiliations:** 1 The Department of Hepatobiliary Surgery, The First Affiliated Hospital, Sun Yat-sen University, Guangzhou, PR China; 2 Liver Cancer Institute, Zhongshan Hospital, Fudan University, Key Laboratory of Carcinogenesis and Cancer Invasion (Fudan University), Ministry of Education, Shanghai, PR China; 3 Cancer Center, Institutes of Biomedical Sciences, Fudan University, Shanghai, PR China; 4 Shanghai Key Laboratory of Organ Transplantation, Shanghai, PR China; Sapporo Medical University, Japan

## Abstract

**Background:**

The aim of this study is to investigate the expression profile of multiple epithelial mesenchymal transition (EMT)-related molecules in intrahepatic cholangiocarcinoma (ICC) and the related prognostic significance.

**Methods:**

Immunohistochemistry was performed to determine the expression of E-cadherin, Vimentin, Snail, slug and β-catenin in a tissue microarray consisting of tumor tissues of 140 ICC patients undergoing curative resection. The correlation between the expression of these molecules and the clinicopathological characteristics of ICC patients was analyzed, and their prognostic implication was evaluated.

**Results:**

Reduced E-cadherin and increased Vimentin expression, the characteristic changes of EMT, identified in 55.0% and 55.7% of primary ICCs, respectively, were correlated with lymphatic metastasis and poorer overall survival (OS) and disease-free survival (DFS) of ICCs. The overexpression of snail and nonmembranous β-catenin, which are the major regulators of the EMT, were identified in 49.2% and 45.7% of primary ICCs, while little slug expression was detected in ICCs. Cytoplasmic/nuclear β-catenin did not significantly predict worse DFS and was not related with E-cadherin loss. The overexpression of snail predicted worse OS and DFS. Snail overexpression correlated with the down-regulation of E-cadherin and the up-regulation of Vimentin. Inhibition of snail in an ICC cell line decreased the expression of E-cadherin, enhanced the expression of Vimentin and impaired the invasion and migration ability of ICC cells.

**Conclusions:**

These data support the hypothesis that EMT plays vital roles in ICC progression and suggest that snail but not slug and β-catenin plays a crucial role in the EMT induction of ICC.

## Introduction

Intrahepatic cholangiocarcinoma (ICC) is a form of aggressive malignancies that arise in the biliary tract and are challenging to diagnose, prevent or treat [1]. However, the tumor heterogeneity and molecular characteristics of ICC are largely unknown.

The epithelial-mesenchymal transition (EMT) is a developmental process in which epithelial cells lose their polarity and acquire the migratory properties of mesenchymal cells. The EMT has been shown to be the pivotal mechanism contributing to the invasiveness and metastatic potential of human cancers, including hepatoma, breast and pancreatic carcinoma [2]. Characteristic alterations that occur during EMT include down-regulation of epithelial markers (e.g., E-cadherin and plakoglobin) and up-regulation of mesenchymal markers (e.g., Vimentin and N-cadherin) [3]. In general, the suppression of E-cadherin expression by the major EMT regulators initiates the EMT process [3]. Snail and slug, zinc-finger transcriptional repressors, are two strong repressors of E-cadherin gene transcription, and the accumulation of Snail and slug in nuclei strongly represses the expression of E-cadherin, which then triggers the EMT of the cancer cells [3], [4]. In addition, some studies have also shown that β-catenin/LEF (lymphoid enhancer binding factor)-1 complex can induce EMT directly when the transcriptional activity of the complex is activated by stable nuclear β-catenin [5], [6]. The translocation of β-catenin to the nucleus leads to a loss of E-cadherin function ^7^. On the other hand, β-catenin binds tightly to the cytoplasmic domain of type I cadherins and plays an essential role in the structural organization and function of cadherins. The E-cadherin cytoplasmic domain interacts with β-catenin or plakoglobin, which in turn bind to α-catenin. α-catenin connects the cadherin-catenin complex to actin filament networks, leading to increased adhesion strength. The loss of E-cadherin results in impairment of cell-cell adhesion, which allows for the detachment of cells and nuclear localization of β-catenin [7].

The clinical significance of the EMT has been established in certain types of human cancers [8], [9]. However, only few sporadic studies have focused on the role of EMT in ICC. Considering the converging data suggesting a role for the EMT during cancer progression, in this study, we initiated an exhaustive screening of 140 clinical samples of ICC to detect links between E-cadherin, Vimentin, snail, slug, β-catenin and tumor progression status.

## Materials and Methods

### Patients and follow-up

Archival specimens were obtained from 140 ICC patients who underwent curative resection between February 1999 and November 2006 at the Liver Cancer Institute, Zhongshan Hospital, Fudan University. Ethical approval was obtained from Zhongshan Hospital Research Ethics Committee, and written informed consent was obtained from each patient. The samples were taken from areas next to the tumor margin. Curative resection was defined as a complete resection of tumor nodules with the cut surface being free of cancer by histological examination; a resection of the regional lymph nodes, including the hilar, hepatoduodenal ligament and caval lymph nodes; and the absence of cancerous thrombus in the portal vein, hepatic veins and bile duct. Follow-up data were collected until February 2009, and the median follow up period was 25 months (range 4–120 months).

### Tissue microarray and immunohistochemistry (IHC)

Tissue microarrays (TMA) were constructed as described in our previous study [10]. Immunohistochemistry was performed as below: the slides were dewaxed by heating at 60°C overnight and washing twice, 10 minutes each, with xylene. The tissues were rehydrated using a series of 5-minute washes with 95%, 80%, 75% ethanol and distilled water. Endogenous peroxidase activity was blocked using 3% hydrogen peroxide for 15 minutes. Samples were heated in 10 mmol/L sodium citrate (pH 6.0) at 95°C for 15 minutes to retrieve the antigen. After blocking with universal blocking serum for 60 minutes, the samples were incubated with relevant antibodies at 4°C overnight. The sections were then incubated with biotin-labeled secondary antibody and streptavidin-peroxidase for 30 minutes each. The samples were developed using 3,3′-diaminobenzidine and lightly counterstained by hematoxylin. The slides were then dehydrated following a standard procedure and sealed with coverslips. Each image was captured using Leica QWin Plus v3 software (Leica Microsystems Imaging Solutions, Cambridge, UK). The antibodies used in immunohistochemistry were as follows: E-cadherin (24E10) mAb (1∶100, Cell Signaling Technology, Beverly, MA, #3195S), snail (A242) pAb (1∶200, Novus biologicals, Littleton, CO, NBP1-19529), slug (C19G7) mAb (1∶100, Cell Signaling Technology, Beverly, MA, #9585P), Vimentin (SP20) mAb (1∶200, Abcam, Cambridge, MA, ab16700) and β-catenin (D10A8) mAb (1∶100, Cell Signaling Technology, Beverly, MA, #8480P).

### Evaluation of immunohistochemical variables

The intensity of positive staining was measured as described previously [11]. The intensity of E-cadherin, snail and slug staining was evaluated using the following criteria: strong staining, dark brown staining in >50% of tumor cells; weak staining, any lesser degree of brown staining appreciable in tumor cell; and absent, no appreciable staining in tumor cells. Only strong staining was considered positive staining. As in previous studies, the tumors were considered positive when Vimentin expression was detected in more than 10% of the tumor cells [12], [13]. For β-catenin protein immunoreaction, the tumors were considered β-catenin nuclear positive when more than 5% of the nuclei of the tumor cells were positively stained [14].

### Cell lines, transfection experiment, wound-healing assay and Matrigel invasion assay

The RBE cell line which derived from intrahepatic cholangiocarcinoma with moderately differentiated tubular adenocarcinoma was obtained from the Chinese Academy of Sciences Shanghai Branch Cell Bank (Shanghai, China) [15], [16]. The cells were maintained in RPMI-1640 (Gibco, Carlsbad, CA, USA) supplemented with 10% fetal bovine serum (Gibco) at 37°C in a 5% CO^2^ humidified atmosphere. Transfection of the siRNAs was performed with Lipofectamine 2000 (Invitrogen, Carlsbad, CA, USA) according to the manufacturer's instructions. The siRNA was constructed and synthesized by Shanghai GeneChem Co., Ltd. The used siRNA sequence for snail was as followed: forward: 5′-GCGAGCUGCAGGACUCUAATT-3′, reverse: 5′-UUAGAGUCCUGCAGCUCGCTT-3′. The wound-healing assay and Matrigel invasion assay were performed as previous described, with slight modified[10].

### Statistical analysis

The data are expressed as the mean ± standard deviation. The χ^2^ test, Fisher's exact probability and Student's t test were used for comparisons between the groups. Overall survival was defined as the interval between ICC resection and death; patients alive at the end of follow up were removed. The time to recurrence was calculated from the ICC resection date to the first radiological evidence of recurrence. Patients with death in the absence of recurrence were excluded when determining the DFS rate. The significance of parameters on survival and DFS probability was evaluated by the Kaplan-Meier method and differences with log-rank test. Cox's proportional hazards regression model was used to analyze the independent prognostic factors. Statistical analyses were performed by SPSS 16.0 for Windows (SPSS, Chicago, IL, USA). P<0.05 was considered statistically significant.

## Results

### Expression of E-cadherin and Vimentin in ICC

**Figure 1:**
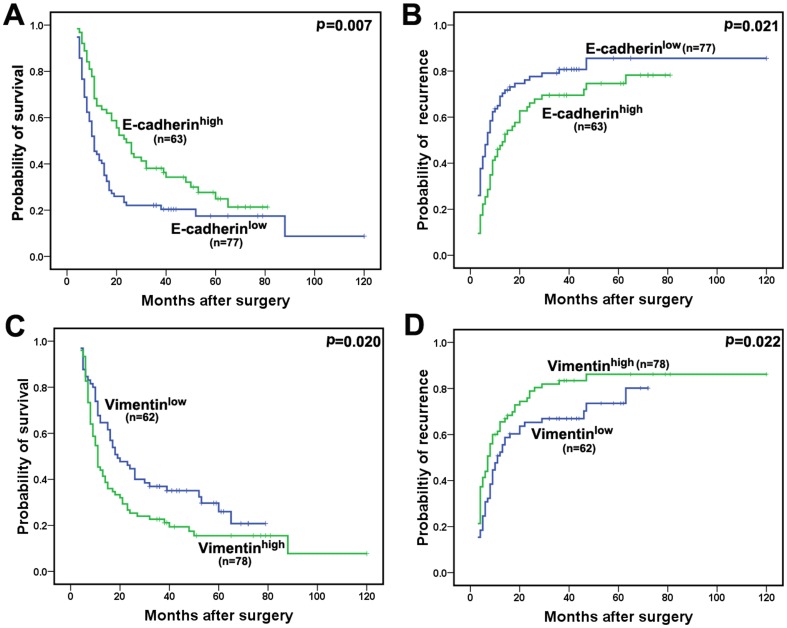
Immunohistochemical analysis of E-cadherin and Vimentin in ICC tissues. (**A, B**) Kaplan-Meier survival analysis of OS and DFS in ICC cases with positive versus negative E-cadherin expression. (**C, D**) Kaplan-Meier survival analysis of OS and DFS in ICC cases with positive versus negative Vimentin expression.

In adjacent nontumorous liver tissues, Interlobular bile ducts showed strong and homogeneous membranous staining (**Figure S1**). In the tumors, three types of distinct staining patterns were observed (strong *vs.* weak and absent, **Figure S1**). We observed lymphatic metastasis more frequently in tumors expressing either weak or absent E-cadherin than in tumors in which E-cadherin expression was strong (P<0.05; **Table S1**). The 2-year and 5-year OS rates in the positive E-cadherin group were significantly higher than the rates in the negative E-cadherin group (49.2% *vs.* 22.1% and 24.9% *vs.* 17.5%, respectively; **Figure 1A**). For the DFS analyses, negative E-cadherin expression was significantly associated with higher recurrence (**Figure 1B**).

No Vimentin expression was observed in the adjacent nontumorous liver tissues (**Figure S2**). Vimentin expression was detected in the tumor stroma region and the tumor cells and varied widely (**Figure S2**). Positive Vimentin staining in fibroblasts, endothelial cells, lymphocytes, and macrophages and negative staining of epithelial cells in non-neoplastic tubules served as “built-in” positive and negative controls, respectively. Tumors were considered positive when Vimentin expression was detected in more than 10% of the tumor cells. Lymphatic metastasis was found to associate with high levels of Vimentin expression (**Table S1**). For the OS and DFS analyses, positive Vimentin expression was significantly associated with shorter survival (**Figure 1C, D**).

### Expression patterns of β-Catenin expression in ICC

**Figure 2:**
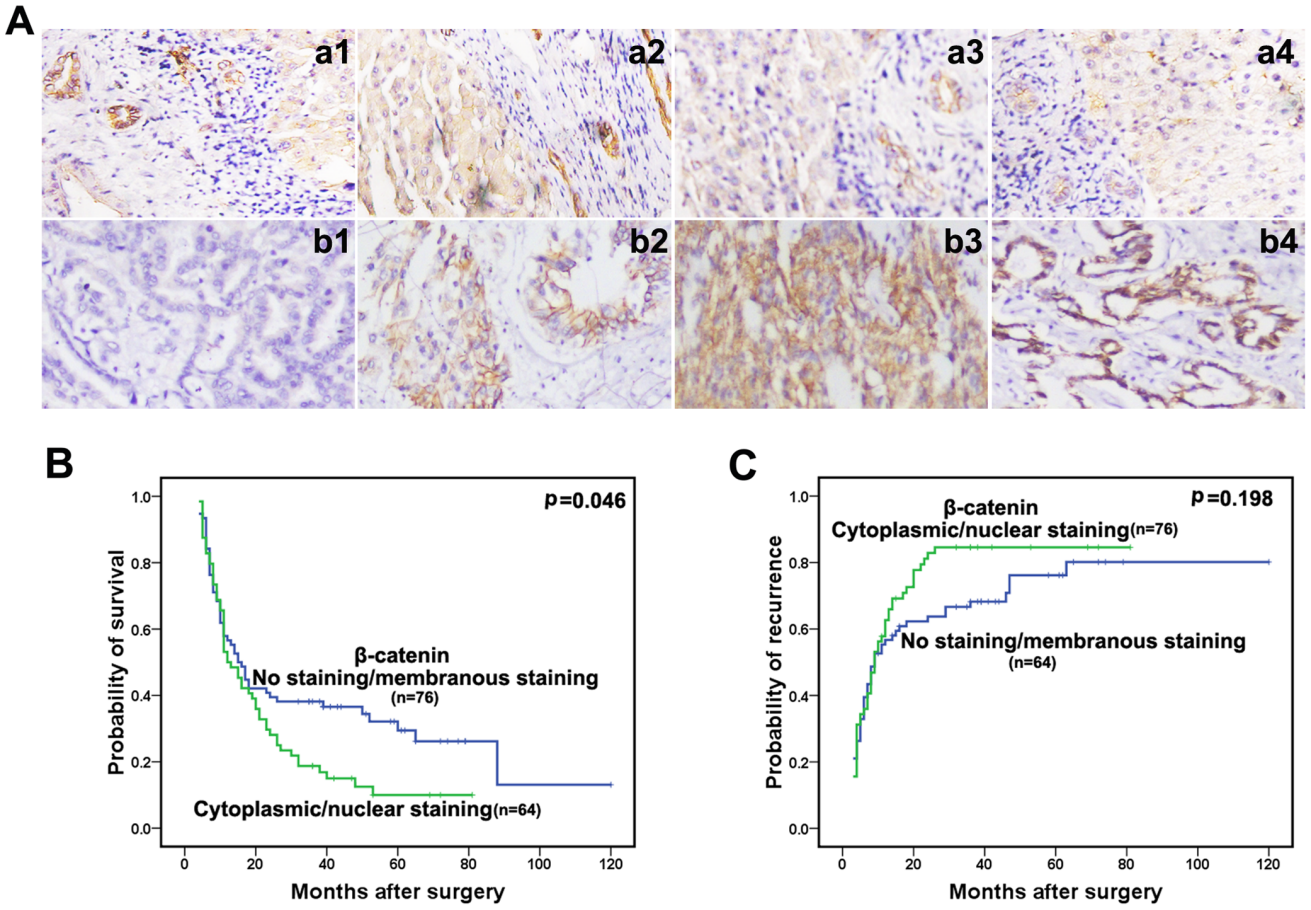
Immunohistochemical analysis of β-catenin in ICC and adjacent nontumorous livers. (**A**) Representative stainings of negative β-catenin (Case 26, a1, b1), membranous β-catenin (Case 78, a2, b2), cytoplasmic β-catenin (Case 11, a3, b3) and nuclear β-catenin (Case 104, a4, b4) in adjacent nontumorous liver tissues (a1, a2, a3, a4) and ICC tissues (b1, b2, b3, b4) were illustrated. Magnification ×200. (**B, C**) Kaplan-Meier survival analysis of OS and DFS in ICCs with negative/membranous expression of β-catenin versus cytoplasmic/nuclear expression of β-catenin expression.

Our observation that enhanced E-cadherin and Vimentin expression were significantly correlated with ICC prognosis raised the question whether the EMT regulators β-catenin, snail and slug might play a similar role in the progression of ICC in humans. The EMT of tumor cells is associated with a nuclear accumulation of the transcriptional activator β-catenin. β-catenin is a major E-cadherin-binding protein at cellular junctions and acts as a key mediator of the Wnt signaling pathway. In the present study, we compared β-catenin immunostaining in 140 ICC patients. Intense staining for β-catenin in nontumorous hepatocytes was mainly located at the membrane (**Figure 2A**). β-catenin expression patterns in tumors were highly variable (**Figure 2A**). Of 140 ICC patients, 76 (54.3%) showed negative or membranous expression of β-catenin, and 64 (45.7%) showed cytoplasmic or nuclear expression of β-catenin. β-catenin expression was not correlated with clinicopathological features (**Table S2**). OS and DFS were estimated by Kaplan-Meier curves. Regarding OS, β-catenin expression was significantly associated with the prognosis of patients with ICC (P = 0.046, **Figure 2B**). In particular, 43.4% of patients in the group with negative or membranous expression of β-catenin died within 12 months after curative resection compared with 50.0% of patients in the group with cytoplasmic or nuclear expression of β-catenin. However, the expression patterns of β-catenin were not related to the DFS of ICC patients (**Figure 2C**, P = 0.198).

Membranous and cytoplasmic β-catenin immunostaining were detected in 14/63 (22%) and 27/63 (43%) tumors, with intense staining at the membrane in tumors expressing positive E-cadherin at the cell surface. Cytoplasmic localization of β-catenin in tumor cells was found in 55 of 140 (39.2%) ICC cases, with nearly equal distribution between E-cadherin-positive and E-cadherin-negative tumors (**Table 1**). Nuclear accumulation of β-catenin was observed in 9 tumors. β-catenin expression patterns were highly heterogeneous in E-cadherin-negative tumors. Only 4 of 77 E-cadherin-negative tumors showed nuclear β-catenin staining, suggesting that E-cadherin loss was not sufficient *per se* for nuclear translocation of unbound β-catenin. These data also suggest that other members of the cadherin family might retain β-catenin in membranous complexes. By contrast, of the 63 tumors in which E-cadherin was present, no cell membrane β-catenin staining could be detected in 49 cases. Tyrosine phosphorylation of E-cadherin/β-catenin complexes mediated by tyrosine kinase might be responsible for the disturbance of junctional E-cadherin/β-catenin complexes in these cases [17], [18].

**Table 1:** Cellular Distribution of β-catenin in ICCs with Different E-cadherin Immunostaining Patterns.

Variables	No staining	Membranous staining	Cytoplasmic staining	Nuclear staining	*P* value
E-Cadherin					
High	17(27%)	14(22%)	27(43%)	5(8%)	0.187
Low	34(44%)	11(14%)	28(36%)	4(5%)	

### Expression and clinical significance of the EMT regulator snail and slug in ICC

**Figure 3:**
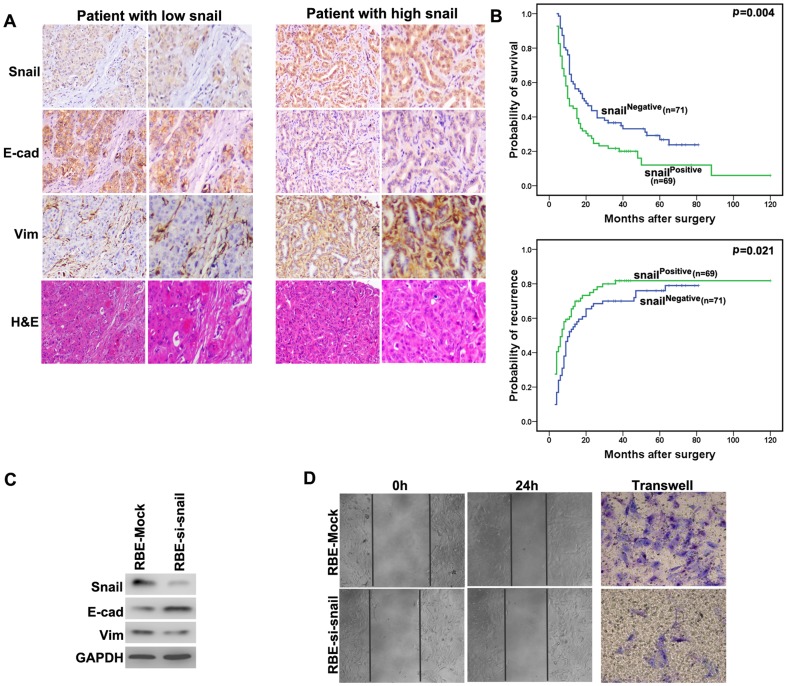
Comparative analysis of snail expression in ICC, and immunostaining of E-cadherin, Vimentin and H&E in corresponding tumors. (**A**) Immunohistochemistry of snail, E-cadherin, Vimentin and H&E in two representative cases without (Case 68, left) or with (Case 36, right) EMT change. (**B**) Kaplan-Meier survival analysis of OS and DFS in ICC cases with high versus low Snail expression. (**C**) The expression of E-cadherin and Vimentin was investigated in RBE-Mock and RBE-si-snail. (**D**) The invasion and migration ability of ICC was investigated in RBE-Mock and RBE-si-snail in vitro.

We examined the expression of snail and slug in 140 ICC patients. Here, we demonstrated that little slug expression was detected in ICC patients, with weak staining in 6 samples (4.3%) and absent in 134 samples (95.7%) (**Figure S3**). However, the snail expression varied in tumor samples. Tumor samples were classified as either positive or negative for snail expression (**Figure 3A**). The relationship between the snail expression level and clinicopathological parameters was then determined. Only lymphatic metastasis was found to associate with high levels of snail expression (**Table S1**). Applying Kaplan-Meier analysis to positive versus negative snail-expressing groups revealed a significant correlation between ICCs that expressed positive levels of snail and OS (P = 0.004). In addition, a statistically significant correlation between positive snail expression and high tumor recurrence was observed within this patient group (P = 0.021, **Figure 3B**).

To establish a putative link between snail and cell phenotypes, we screened ICC tissues for cell-cell adhesion status, which are reliable markers for epithelial and mesenchymal phenotype. Studies included IHC for snail, E-cadherin and Vimentin. An increased incidence of Snail overexpression has been shown in recurrent versus non-recurrent ICC. For each tumor, snail expression was plotted versus the expression of E-cadherin and Vimentin (**Figure 3A**). Of the 63 tumors that showed negative E-cadherin expression, 45 (71%) of them were positive for Snail expression. The analysis of 140 ICCs showed a clear negative correlation between snail and E-cadherin expression (**Table 2**, P<0.001). ICC also exhibited a positive correlation between snail and Vimentin expression (**Table 2**, P = 0.037). We sorted the cells by increasing the expression level of snail to observe a putative link between snail expression levels and phenotypic groups. As expected, tissues with positive snail expression had a high proportion of cells with a mesenchymal phenotype (**Figure 3A**).

**Table 2:** Expression of snail in ICCs with Different E-Cadherin and Vimentin Immunostaining Patterns.

Variables	High snail	Low snail	*P* value
E-cadherin			
High	18(29%)	45(71%)	<0.001
Low	50(65%)	27(35%)	
Vimentin			
High	44(56%)	34(44%)	0.037
Low	24(39%)	38(61%)	

Since we had observed a correlation between snail expression and acquisition of a mesenchymal phenotype in ICC tissues, we wished to test whether snail could induce EMT in vitro. Here, we interfered the expression of snail in ICC cell line RBE, and we demonstrated that the level of E-cadherin was higher in RBE-si-snail than in RBE-Mock, while Vimentin was down-regulated in RBE-si-snail cells (**Figure 3C**). Moreover, Matrigel invasion assays revealed that decreased snail expression was accompanied by impairment in the invasiveness of RBE cells. A wound-healing assay revealed an evident delay in the wound closure rate of RBE-si-snail cells at 24 hours compared with RBE-Mock cells (**Figure 3D**). So we conclude snail play an important role in the EMT induction of ICC.

## Discussion

Although the EMT has been considered a critical mechanism involved in cancer metastasis, only few sporadic studies have focused on the role of EMT in ICC [19]. Here, our study reinforces the hypothesis that the EMT process enables epithelial cancer cells to achieve invasion and metastasis. First, we detected that the reduced expression of E-cadherin correlated with poor prognosis. Next, we observed that Vimentin expression in ICC was linked originally to lymph node metastasis and associated with poor prognosis of ICC. We also detected expression of another mesenchymal marker, N-cadherin in ICC and found that up-regulation of N-cadherin was significantly associated with a poor outcome (data not shown). As to the inducer of the EMT, we found that snail expression was related to poor prognosis in ICC. Importantly, our results revealed that snail expression was positively correlated with Vimentin and negatively correlated with E-cadherin, and inhibition of snail in vitro decreased the expression of E-cadherin, enhanced the expression of Vimentin and impaired the invasion and migration ability of ICC cells. We maked the point that β-catenin was not considered to be negative with E-cadherin expression in ICC. As for slug, little expression was detected in ICC, so it is excluded for further investigation.

In the present study, we provide evidence that the down-regulation of epithelial markers (E-cadherin) and up-regulation of mesenchymal markers (Vimentin) could represent characteristic changes during the EMT: negative E-cadherin expression and positive Vimentin expression indicates worse prognoses in ICC patients. The EMT or loss of differentiation is frequently observed and is likely to mediate cellular detachment and eventual metastasis. In many carcinoma cell lines, the induction of EMT results in the acquisition of metastatic properties in vivo [20], and metastasis is in accord with partial or complete EMT. More recently, several studies have demonstrated that cancer cells undergoing EMT appear to gain stem cell properties, contribute to immunosuppression, and prevent senescence [21]–[23], which further underscores the cardinal manifestation of the EMT in tumor metastasis.

The EMT process is thought to be initiated through the suppression of E-cadherin expression by major EMT regulators, such as snail overexpression. The analysis of 140 ICCs showed a clear negative correlation between snail and E-cadherin expression (P<0.001). ICCs also exhibited a positive correlation between snail and Vimentin expression (P = 0.037). Given the strong repression of E-cadherin that was observed in snail-expressing tumor samples, the well-described association of E-cadherin with poor prognosis may, in some cases, reflect snail activity. Indeed, an inverse association between snail and E-cadherin expression has been reported in some human cancers [24]. Inflammation is a high risk factor for the development of ICC and activation of NF-kB in inflammatory response promote the malignant transformation of normal cells [25]. Expression of Snail correlate with the activation of NF-kB and knockdown of Snail expression significantly inhibit cell migration and invasion induced by inflammation [26], [27]. In this research, we chose NF-kB subunit, p65 to study and found that the expression of p65 is positively correlated with Snail in ICC patients (**Figure S4**). However, the regulatory mechanism and interaction of p65 and Snail is still unclear, we will take this question as focus and take a further investigation. Our data address which of snail's many functions are most relevant to the recurrence or maintenance of the residual disease. Snail may thereby represent an important new target for the generation of cancer therapeutics directed against specific molecules involved in ICC recurrence.

In ICC, including E-cadherin-positive and E-cadherin-negative expression cases, β-catenin was strongly expressed at the cell membrane, and nuclear accumulation of this protein was not elevated with E-cadherin loss, suggesting that E-cadherin loss was not sufficient *per se* for nuclear translocation of unbound β-catenin. Several studies demonstrated that E-cadherin suppresses cellular transformation by inhibiting β-catenin signaling in an adhesion-independent manner and that E-cadherin negatively regulates β-catenin transcriptional activity by recruiting β-catenin from β-catenin-LEF/TCF (T cell factor) transcriptional complexes, sequestering β-catenin from the cytosol [28], [29]. Clinical evidence has shown that β-Catenin accumulated in the cytoplasm when the expression of E-cadherin was decreased in cancers such as non-small cell lung cancer [30]. However, in our series, no specific IHC pattern of β-catenin expression correlated with E-cadherin in ICC. The relationship between β-catenin and E-cadherin appeared to be context-specific. These discrepancies are most likely due to the complex interactions between factors of the Wnt-signaling pathway, all of which may influence the subcellular localization of β-catenin. In the current study, β-catenin expression was not related with DFS. However, the predictive ability of snail overexpression was more sensitive than that of β-catenin expression, lending support to the hypothesis that snail might play a more important role in the induction of the EMT in ICC than β-catenin.

The present study is the first to provide a comprehensive profile of multiple EMT markers and establishes different roles for snail, slug and β-catenin in ICC. These results pinpoint the major regulator of the induction of the EMT in ICC. These results provide essential information for the prediction of prognosis and for the identification of new potential therapeutic targets for future ICC management.

## Supporting Information

Figure S1 Expression of E-cadherin is illustrated in ICC tissues and adjacent nontumorous tissues. Interlobular bile ducts showed strong and homogeneous membranous staining. E-cadherin is faintly expressed at the hepatocyte membrane in adjacent nontumorous liver tissues (a1, a2, a3). (b1)E-cadherin expression is undetectable in Case 64. (b2) Heterogeneous expression of E-cadherin in Case 37, with strong staining in part of tumor tissues and loss in other areas. (b3) Strong E-cadherin expression in Case 28. Magnification ×200.
(TIF)

Figure S2 Immunohistochemical analysis of Vimentin in ICC and adjacent nontumorous tissues. (A) No Vimentin expression was observed in the adjacent nontumorous liver cells. (B) Vimentin expression was detected in the tumor stroma region. (C, D) Vimentin expression in ICC varied widely. Represented cases were listed. (Positive Vimentin expression: Case 34, Negative Vimentin expression: Case 31) Magnification ×200.
(TIF)

Figure S3 Representative staining of slug in the ICC patients was illustrated.
(TIF)

Figure S4 Immunohistochemical analysis of p65 and snail in ICC tumor tissues. Representative stains (weak: A, B; moderate: C, D; strong: E, F) of p65 and snail from three tumor samples are shown.
(TIF)

Table S1 Correlations between E-cadherin, Vimentin, snail and clinicopathological features in 140 ICC cases.
(DOCX)

Table S2 Correlations between β-catenin and clinicopathological features in 140 ICC cases.
(DOC)
