# Supplementary figures and images for: Comprehensive Multiple Molecular Profile of Epithelial Mesenchymal Transition in Intrahepatic Cholangiocarcinoma Patients
Source: PLoS One. 2014 May 9;9(5):e96860. doi: 10.1371/journal.pone.0096860 (PMC4016113; doi:10.1371/journal.pone.0096860)

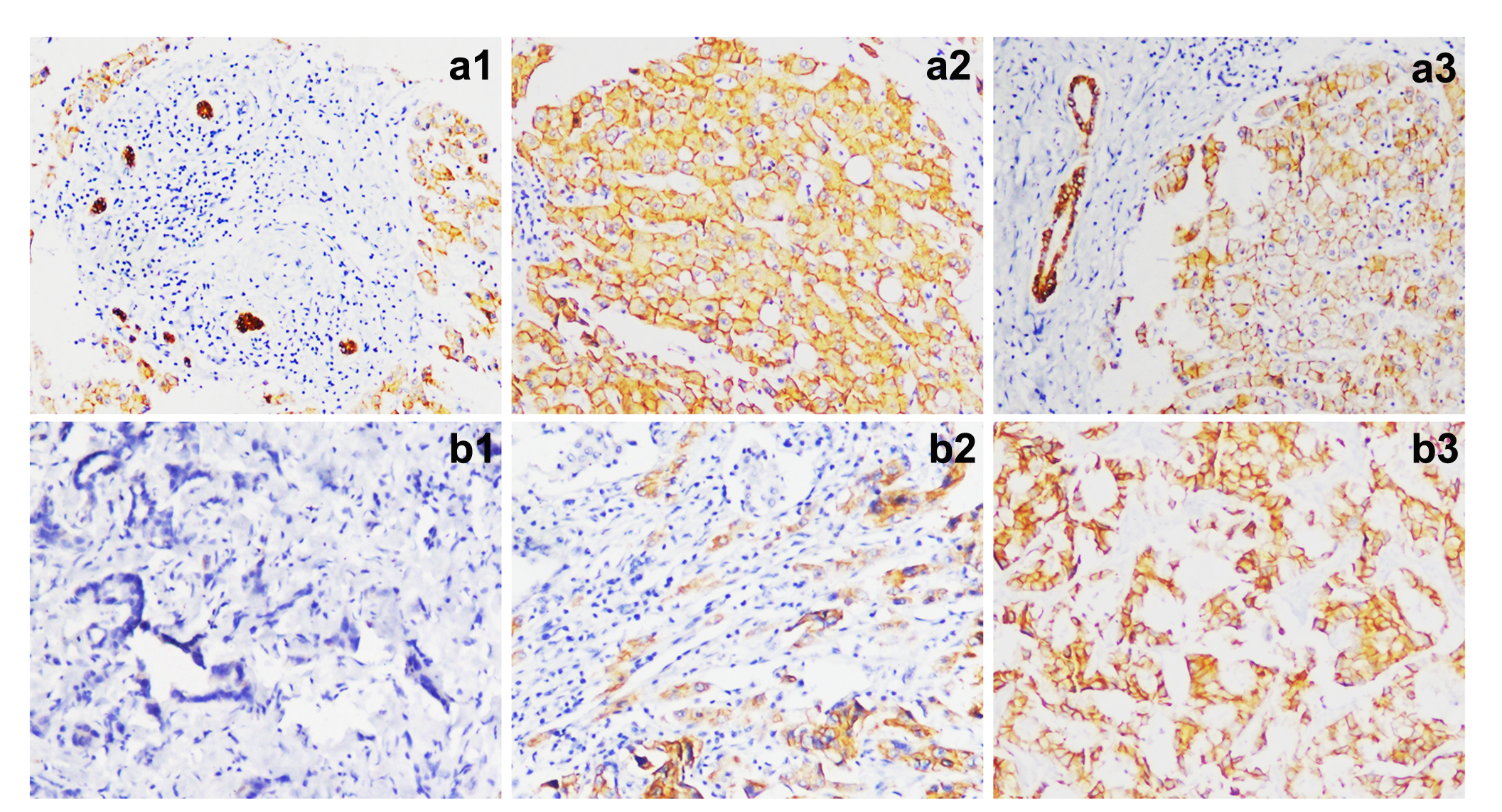

Supplement: Figure S1 — Expression of E-cadherin is illustrated in ICC tissues and adjacent nontumorous tissues. Interlobular bile ducts showed strong and homogeneous membranous staining. E-cadherin is faintly expressed at the hepatocyte membrane in adjacent nontumorous liver tissues (a1, a2, a3). (b1)E-cadherin expression is undetectable in Case 64. (b2) Heterogeneous expression of E-cadherin in Case 37, with strong staining in part of tumor tissues and loss in other areas. (b3) Strong E-cadherin expression in Case 28. Magnification ×200. (TIF) [file pone.0096860.s001.tif]

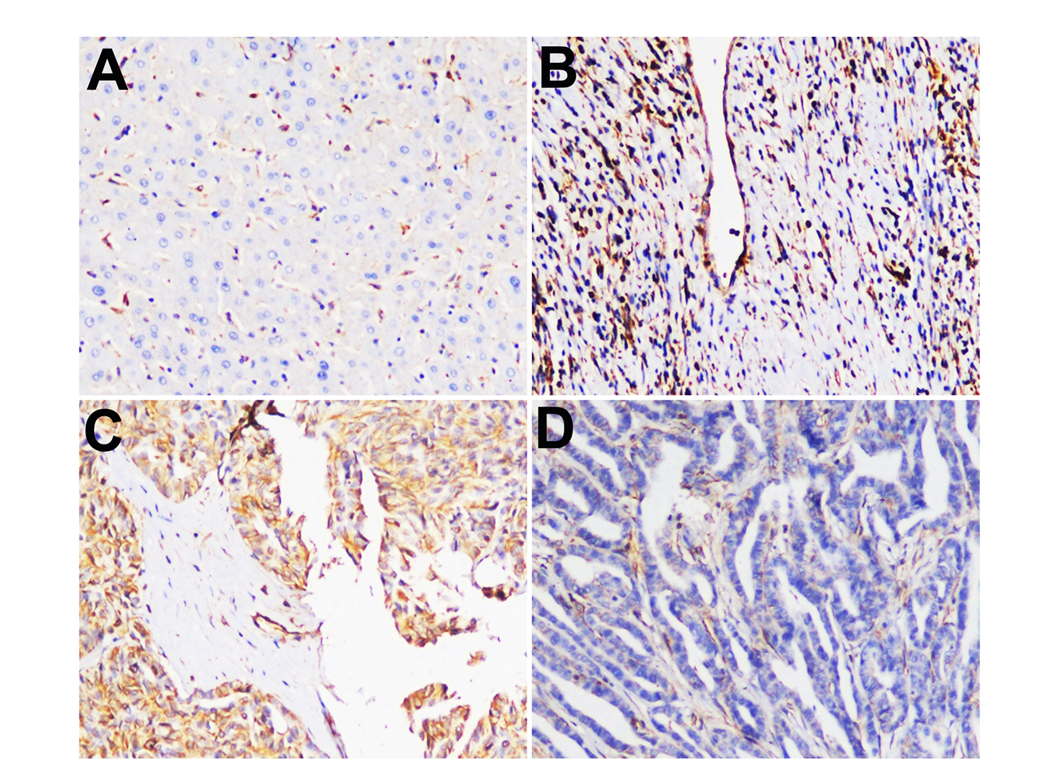

Supplement: Figure S2 — Immunohistochemical analysis of Vimentin in ICC and adjacent nontumorous tissues. (A) No Vimentin expression was observed in the adjacent nontumorous liver cells. (B) Vimentin expression was detected in the tumor stroma region. (C, D) Vimentin expression in ICC varied widely. Represented cases were listed. (Positive Vimentin expression: Case 34, Negative Vimentin expression: Case 31) Magnification ×200. (TIF) [file pone.0096860.s002.tif]

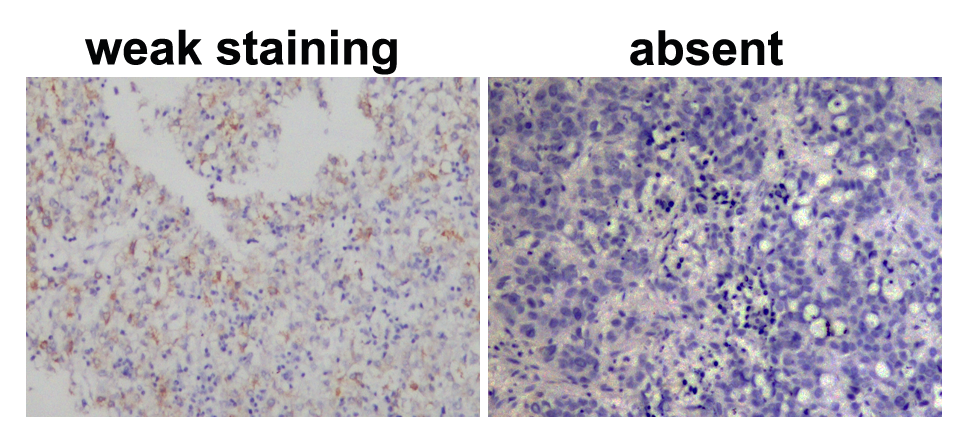

Supplement: Figure S3 — Representative staining of slug in the ICC patients was illustrated. (TIF) [file pone.0096860.s003.tif]

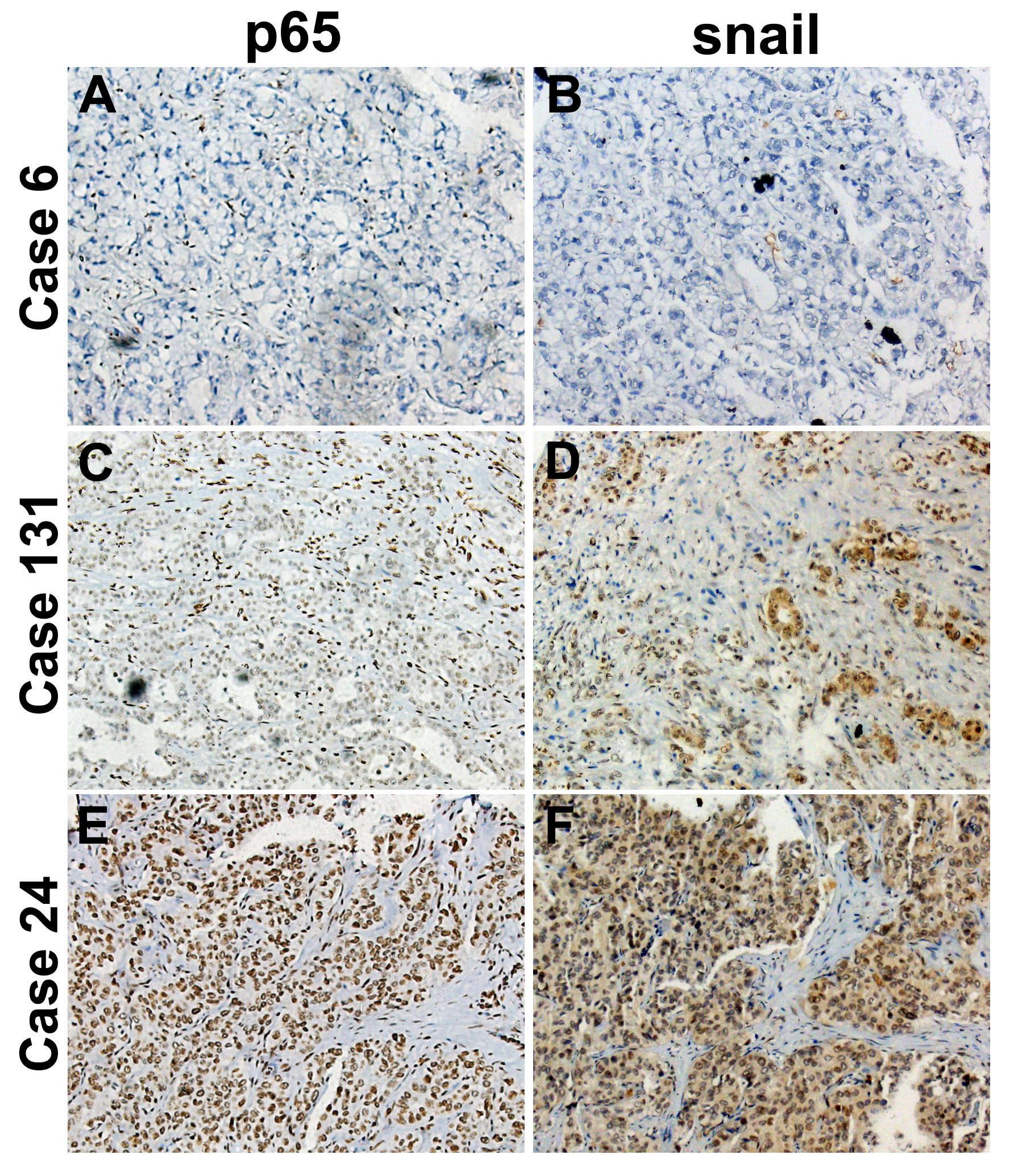

Supplement: Figure S4 — Immunohistochemical analysis of p65 and snail in ICC tumor tissues. Representative stains (weak: A, B; moderate: C, D; strong: E, F) of p65 and snail from three tumor samples are shown. (TIF) [file pone.0096860.s004.tif]
