# Supplementary material for: Comprehensive Multiple Molecular Profile of Epithelial Mesenchymal Transition in Intrahepatic Cholangiocarcinoma Patients
Source: PLoS One. 2014 May 9;9(5):e96860. doi: 10.1371/journal.pone.0096860 (PMC4016113; doi:10.1371/journal.pone.0096860)
Supplement: Table S1 — Correlations between E-cadherin, Vimentin, snail and clinicopathological features in 140 ICC cases. (DOCX) [file pone.0096860.s005.docx]

**Table S1 Correlations between E-cadherin, Vimentin, snail and** **clinicopathological features in 140 ICC cases**

| Variables | E-cadherin | | *P* value | Vimentin | | *P* value | Snail | | *P* value |
| --- | --- | --- | --- | --- | --- | --- | --- | --- | --- |
|  | High  (n=63) | Low  (n=77) |  | High  (n=78) | Low  (n=62) |  | High  (n=69) | Low  (n=71) |  |
| Age (years) | | | | | | | | | |
| ≥53 | 31 | 39 | 0.865 | 38 | 32 | 0.734 | 37 | 33 | 0.398 |
| <53 | 32 | 38 |  | 40 | 30 |  | 32 | 38 |  |
| Sex | | | | | | | | | |
| Male | 29 | 30 | 0.399 | 38 | 21 | 0.077 | 33 | 26 | 0.179 |
| Female | 34 | 47 |  | 40 | 41 |  | 36 | 45 |  |
| HBsAg | | | | | | | | | |
| Positive | 39 | 48 | 0.958 | 50 | 37 | 0.592 | 47 | 40 | 0.151 |
| Negative | 24 | 29 |  | 28 | 25 |  | 22 | 31 |  |
| Serum CA19-9 (ng/ml) | | | | | | | | | |
| ≥37 | 39 | 46 | 0.794 | 47 | 38 | 0.901 | 45 | 40 | 0.282 |
| <37 | 24 | 31 |  | 31 | 24 |  | 24 | 31 |  |
| Child-Pugh score | | | | | | | | | |
| A | 62 | 72 | 0.223^*^ | 73 | 61 | 0.227^*^ | 64 | 70 | 0.113^*^ |
| B | 1 | 5 |  | 5 | 1 |  | 5 | 1 |  |
| Serum AFP (ng/ml) | | | | | | | | | |
| <20 | 57 | 65 | 0.287 | 69 | 53 | 0.601 | 58 | 64 | 0.282 |
| ≥20 | 6 | 12 |  | 9 | 9 |  | 11 | 7 |  |
| Tumor size (diameter, cm) | | | | | | | | | |
| ≤5 | 50 | 59 | 0.698 | 62 | 47 | 0.602 | 54 | 55 | 0.910 |
| >5 | 13 | 18 |  | 16 | 15 |  | 15 | 16 |  |
| Tumor differentiation | | | | | | | | | |
| III/IV | 29 | 39 | 0.587 | 42 | 26 | 0.161 | 34 | 34 | 0.870 |
| I/II | 34 | 38 |  | 36 | 36 |  | 35 | 37 |  |
| Tumor number | | | | | | | | | |
| Multiple | 6 | 5 | 0.507 | 6 | 5 | 0.475 | 5 | 6 | 0.791 |
| Single | 57 | 72 |  | 73 | 56 |  | 64 | 65 |  |
| Microvascular/bile duct invasion | | | | | | | | | |
| Yes | 8 | 15 | 0.281 | 14 | 9 | 0.586 | 11 | 12 | 0.878 |
| No | 55 | 62 |  | 64 | 53 |  | 58 | 59 |  |
| Lymphatic metastasis | | | | | | | | | |
| Yes | 10 | 24 | 0.036 | 25 | 9 | 0.016 | 23 | 11 | 0.014 |
| No | 53 | 53 |  | 53 | 53 |  | 46 | 60 |  |

Abbreviations and Note: ICC, intrahepatic cholangiocarcinoma; AFP, alpha-fetoprotein; *Fisher exact test.
