# Supplementary material for: Comprehensive Multiple Molecular Profile of Epithelial Mesenchymal Transition in Intrahepatic Cholangiocarcinoma Patients
Source: PLoS One. 2014 May 9;9(5):e96860. doi: 10.1371/journal.pone.0096860 (PMC4016113; doi:10.1371/journal.pone.0096860)
Supplement: Table S2 — Correlations between β-catenin and clinicopathological features in 140 ICC cases. (DOC) [file pone.0096860.s006.doc]

**Table S2 Correlations between β-catenin and clinicopathological features in 140 ICC cases**

| Variables | No staining | Membranous staining | Cytoplasmic staining | Nuclear staining | *P* value |
| --- | --- | --- | --- | --- | --- |
| Age(years) | | | | | |
| ≥53 | 30 | 11 | 25 | 4 | 0.473 |
| <53 | 21 | 14 | 30 | 5 |
| Sex | | | | | |
| Male | 23 | 8 | 25 | 3 | 0.621 |
| Female | 28 | 17 | 30 | 6 |
| HBsAg | | | | | |
| Positive | 34 | 12 | 34 | 7 | 0.320 |
| Negative | 17 | 13 | 21 | 2 |
| Serum CA19-9 (ng/ml) | | | | | |
| ≥37 | 31 | 16 | 34 | 4 | 0.767 |
| <37 | 20 | 9 | 21 | 5 |
| Child-Pugh score | | | | | |
| A | 47 | 24 | 54 | 9 | 0.424 |
| B | 4 | 1 | 1 | 0 |
| Serum AFP (ng/ml) | | | | | |
| <20 | 48 | 18 | 48 | 8 | 0.061 |
| ≥20 | 3 | 7 | 7 | 1 |
| Tumor size (diameter, cm) | | | | | |
| ≤5 | 38 | 20 | 45 | 6 | 0.670 |
| >5 | 13 | 5 | 10 | 3 |
| Tumor differentiation | | | | | |
| III/IV | 24 | 9 | 29 | 6 | 0.363 |
| I/II | 27 | 16 | 26 | 3 |
| Tumor number | | | | | |
| Multiple | 3 | 2 | 5 | 1 | 0.914 |
| Single | 48 | 23 | 50 | 8 |
| Microvascular/ bile duct invasion | | | | | |
| Yes | 8 | 5 | 8 | 2 | 0.893 |
| No | 43 | 20 | 47 | 7 |
| Lymphatic metastasis | | | | | |
| Yes | 14 | 4 | 15 | 1 | 0.540 |
| No | 37 | 21 | 40 | 8 |

Abbreviations and Note: ICC, intrahepatic cholangiocarcinoma; AFP, alpha-fetoprotein; HBsAg, hepatitis B surface antigen; X^2^ test. ^＊^Fisher’s Exact Test
